# A comprehensive transferability evaluation of U-Net and ResU-Net for landslide detection from Sentinel-2 data (case study areas from Taiwan, China, and Japan)

**DOI:** 10.1038/s41598-021-94190-9

**Published:** 2021-07-16

**Authors:** Omid Ghorbanzadeh, Alessandro Crivellari, Pedram Ghamisi, Hejar Shahabi, Thomas Blaschke

**Affiliations:** 1 Department of Geoinformatics—Z_GIS, University of Salzburg, 5020 Salzburg, Austria; 2 Machine Learning Group, Helmholtz-Zentrum Dresden-Rossendorf, Helmholtz Institute Freiberg for Resource Technology, Chemnitzer Str. 40, 09599 Freiberg, Germany; 3 Institute of Advanced Research in Artificial Intelligence (IARAI), Landstraβer Hauptstraβe 5, 1030 Vienna, Austria; 4 Institute of Geography, Slovak Academy of Sciences, Stefanikova 49, 814 73 Bratislava, Slovakia

**Keywords:** Natural hazards, Hydrogeology

## Abstract

Earthquakes and heavy rainfalls are the two leading causes of landslides around the world. Since they often occur across large areas, landslide detection requires rapid and reliable automatic detection approaches. Currently, deep learning (DL) approaches, especially different convolutional neural network and fully convolutional network (FCN) algorithms, are reliably achieving cutting-edge accuracies in automatic landslide detection. However, these successful applications of various DL approaches have thus far been based on very high resolution satellite images (e.g., GeoEye and WorldView), making it easier to achieve such high detection performances. In this study, we use freely available Sentinel-2 data and ALOS digital elevation model to investigate the application of two well-known FCN algorithms, namely the U-Net and residual U-Net (or so-called ResU-Net), for landslide detection. To our knowledge, this is the first application of FCN for landslide detection only from freely available data. We adapt the algorithms to the specific aim of landslide detection, then train and test with data from three different case study areas located in Western Taitung County (Taiwan), Shuzheng Valley (China), and Eastern Iburi (Japan). We characterize three different window size sample patches to train the algorithms. Our results also contain a comprehensive transferability assessment achieved through different training and testing scenarios in the three case studies. The highest f1-score value of 73.32% was obtained by ResU-Net, trained with a dataset from Japan, and tested on China’s holdout testing area using the sample patch size of 64 × 64 pixels.

## Introduction

Landslides have significant direct and indirect adverse effects on the natural environment and resources of large areas, lead to economic loss in the local communities by damaging property and infrastructure, cause fatalities, and affect areas worldwide^1^. In recent years, landslides have become even more frequent and harmful because of climate change, population growth, and unplanned urbanization in mountainous areas, which are very dynamic in terms of sedimentation and erosion^2,3^. A range of human-induced and/or natural triggers, such as road construction, earthquakes, volcanic eruptions, rapid snowmelt, and heavy rainfalls can initiate mass movements and natural hazard cascades by damming streams and causing catastrophic flash floods and debris flows^4–6^. Timely landslide detection and inventory mapping are vital to enable fast delivery of humanitarian aid and crisis response^7^. Moreover, accurate landslide detection to obtain spatial information on landslides, including their exact location and extent, is a prerequisite for any further analysis, such as susceptibility modelling, risk evaluation, and vulnerability assessments^8,9^.

Remote Sensing (RS) images play an essential role in gaining a deeper and more complete understanding of the precise locations, boundaries, extents, and distributions of landslides^10–12^. Therefore, landslide inventory maps are usually prepared by extracting the landslide information from RS images, including optical satellite images and synthetic aperture radar (SAR) data, because of the relatively low cost associated with obtaining RS images and their wide coverage area^13,14^. There is a range of common landslide inventory mapping approaches using optical satellite images, such as the manual extraction of landslide areas based on an expert’s visual interpretation, rule-based image classification approaches carried out by an experienced analyst^1,15^, analyzing of the multi-temporal SAR interferometry techniques^16^, applying optical or LiDAR data from unmanned aerial vehicles^17,18^ and the semi-automatic/ automatic image classification using Machine Learning (ML) models in both pixel- and object-based working environments^19,20^.

Generally, applications of knowledge-based or ML (e.g., K-means clustering) models in the pixel-based working environment are typically based on the spectral information of every single pixel in the RS image^21^. Therefore, pixel-based approaches fail to consider the geometric and contextual information of the features represented in the RS image and have a salt-and-pepper problem in the resulting maps, which requires a lot of post-processing in manual corrections^22^. For over two decades, Geographic Object-Based Image Analysis (GEOBIA)^23^ has been used for landslide monitoring^24^ and detection by grouping similar pixels into meaningful objects and merging objects with related spectral, textural, shape, context, and topological properties^25^. Bacha et al.^26^ evaluated the performance and transferability of the threshold values of some of these parameters. It concluded that they are not directly transferable to other study areas and datasets. Therefore, expert knowledge and analyst experience play a vital role in determining the properties and parameters associated with a particular case study to achieve the desired accuracy of the resulting landslide inventory map^24,27^.

During the past decade, Deep Learning (DL) approaches, and especially different algorithms of Convolutional Neural Network (CNN) and Fully Convolutional Network (FCN), have been steadily optimized to achieve state-of-art results in RS image classification^28–30^. DL approaches involve extracting features from the input RS images using the convolutional layers and then detecting landslide areas by learning high and low-level features. In recent years, a few studies have used DL approaches for landslide detection. Chen et al.^31^ used a CNN algorithm based on the Gaofen-1 High-Resolution (HR) RS image and slope information derived from a 5 m spatial resolution digital elevation model (DEM) for landslide extraction in three different cities in China. They used 28 × 28 pixels sample patches and achieved a quality percentage of 61%. Ghorbanzadeh et al. 2019 evaluated two different CNN algorithms trained by different sample patches with a range of window sizes from 12 × 12 to 48 × 48 pixels and compared the landslide detection results with those of state-of-art Machine Learning (ML) models. Using RapidEye 5 m spatial resolution imagery along with a 5 m resolution DEM resulted in the highest mean intersection-over-union (mIOU) of more than 78% in their study. The same CNN algorithms were then compared to the Residual Networks (ResNets) by^32^ for landslide detection in Malaysia. They achieved the highest f1-score of 90% and a mIOU of more than 90% using a ResNets algorithm, followed by a CNN, which resulted in a f1-score of 83% and a mIOU of 83.27%. All approaches were trained and tested based on a database including aerial photographs and a DEM with 1 m spatial resolution and 10 cm ground accuracy. Shi et al.^20^ also applied a CNN algorithm for landslide recognition in Hong Kong based on aerial photographs with a 0.5 m spatial resolution. Using a post-processing method for mask operations and screening, they were able to achieve the highest accuracy of more than 80%.

This year, different FCN algorithms also received much attention from researchers for RS image classification, and a limited number of studies evaluated these algorithms for landslide detection. Soares et al.^33^ used a RapidEye image (5 m spatial resolution) and an ALOS DEM for training and testing a U-Net for landslide detection in the mountainous region of Rio de Janeiro, Brazil. Different sample patch window sizes were used in their study, and the best result was based on 128 × 128 pixels with an f1-score of 55%. Liu et al*.*^34^ also trained and tested a U-Net on two optical imageries with different spatial resolutions of 0.14 m and 0.47 m, respectively. They achieved very high accuracy of over 91% for their case study area in Northern Sichuan Province. Qi et al*.*^35^ compared the performance of the ResU-Net with that of the normal U-Net for detecting regional landslides in a semi-arid region in Gansu Province, China. Using the GeoEye-1 image with a nominal spatial resolution of 0.5 m, the F1 metric of landslide detection was 80% for the U-Net and 89% for the ResU-Net. Su et al*.*^36^ also used the U-Net in different scenarios for landslide detection from bitemporal RGB aerial images and a DTM that was obtained from airborne LiDAR data. The spatial resolution of both the RGB aerial images and the DTM was 0.5 m.

The FCN algorithms and their variations also applied to Sentinel-2 imageries for some other purposes. Masoud et al.^37^, for instance, investigated the delineation of agricultural field boundaries from Sentinel-2 imageries, developing a novel FCN algorithm. The accuracy of their resulting delineation maps was slightly lower than those of from RapidEye images acquired at 5 m resolution.

Our literature review indicates that various CNN and FCN algorithms have been used for landslide detection. Moreover, although the developed/applied algorithms have achieved state-of-the-art baselines in landslide detection, there has not been one study that used a DL approach based on freely available satellite data. All the studies were done based on HR and /or Very High Resolution (VHR) satellite imageries (e.g., GeoEye and WorldView). The spatial resolution of the applied RS images in the mentioned studies ranges from 0.14 to 5 m, which is a considerable factor in the spatial heterogeneity and achieving such high accuracies. However, acquiring such VHR satellite imageries is usually expensive, is not available everywhere, and has less temporal observation frequency.

This study’s main objective is to showcase the potential of a well-known FCN algorithm of the U-Net for landslide detection from freely available Sentinel-2 data and ALOS DEM and compare it with the results of ResU-Net. We also evaluate the impact of applying different sample patch window sizes on the detection results. Moreover, we assess the transferability performance of the applied approaches using three case study areas in Western Taitung County (Taiwan), Shuzheng Valley (China), and Eastern Iburi (Japan). The results are then validated using common RS validation metrics, namely precision, recall, and f1-score.

## Materials and methods

### Study areas and inventory maps

#### Eastern Iburi (Japan)

**Figure 1:**
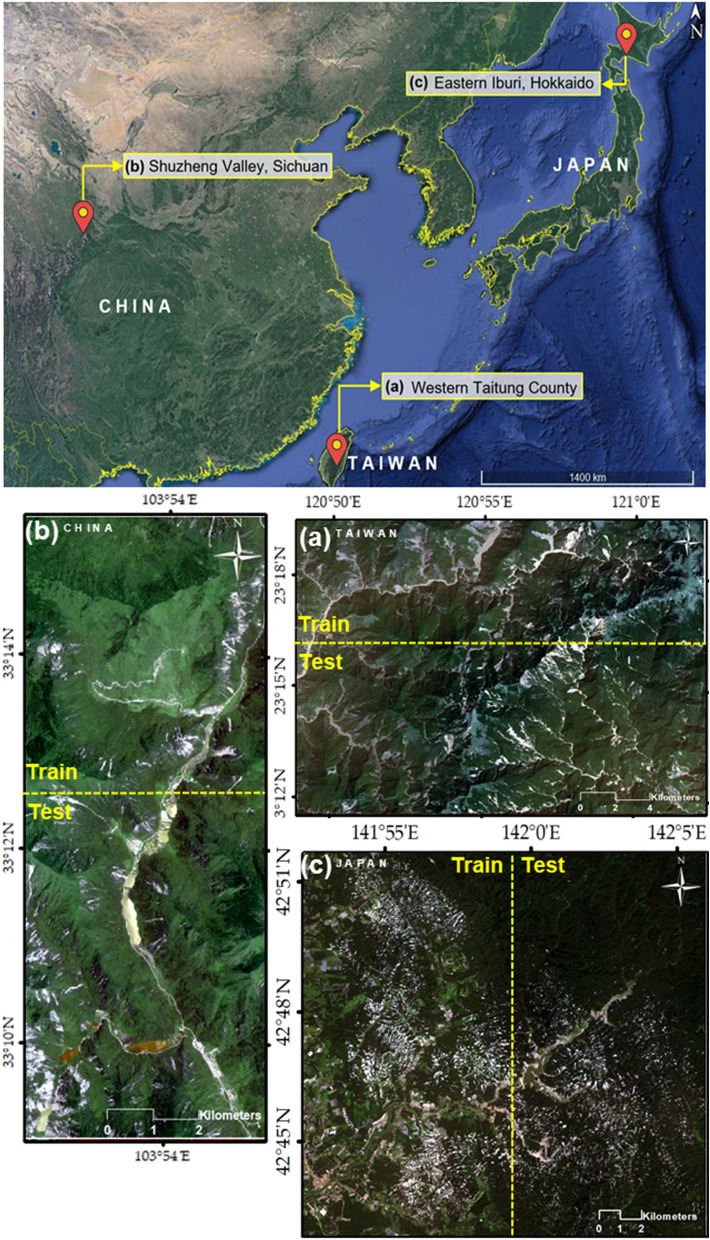
Overview of the study areas: (**a**) Shuzheng Valley (China); (**b**) Western Taitung County (Taiwan); and (**c**) Eastern Iburi (Japan). The training and testing areas are presented on Sentinel-2 images, band combination 1–2–3 (https://scihub.copernicus.eu/). The upper figure was drawing based on Google Earth and (**a**), (**b**), and (**c**) were generated with the ArcMap v.10.8 software (https://desktop.arcgis.com/es/arcmap/).

On September 6, 2018, an earthquake with a magnitude (Mw) of 6.6 struck Eastern Iburi, Hokkaido, Japan. It resulted in widespread destruction, including power cuts, the destruction of power distribution networks, and damage to the Tomato-Atsuma Power Station, which provides electricity for Hokkaido Island^38,39^. Furthermore, the earthquake caused several deep-seated and shallow landslides. Of the 41 deaths related to this earthquake, 36 were due to landslides^40^. Following the earthquake, almost 5600 landslides occurred near Atusma town. The main reason for the significant number of landslides was typhoon Jebi, which brought torrential rainfalls to the region the day before the earthquake and soaked the region’s subsurface, making it more prone to landslides^40,41^. Since the depth of the surface soil layer varies from 4 to 5 m, most of the landslides were shallow and primarily affected the hilly regions between the elevations of 200 and 400 m^38^. In addition to shallow landslides, the area was also affected by planar and spoon-type deep-seated landslides^41^. The landslide inventory map in this region is generated by the Geographical Survey Institute (GSI) of Japan using aerial ortho-photographs^42^. Zhang et al.^43^ updated the landslide inventory map by performing spatial analyses on VHR aerial images and a 10-m resolution DEM. Therefore, 5625 features were reported as landslides over an area of 46.3 km^2^. In this study, we used their landslide geodatabase for Eastern Iburi in ESRI shape format, and then extracted all landslides (Almost 4940 features with a total area of 43.17 km^2^) within our pre-defined study area (Fig. 1C) in Eastern Iburi. The maximum, minimum, and mean of the landslide features are 569,904.02 m^2^, 89.6 m^2^, and 8688.8 m^2^ respectively.

#### Western Taitung county (Taiwan)

Natural hazards such as earthquakes, floods, and landslides are common phenomena in Taiwan^44^. In August 2009, Morakot, the deadliest typhoon in Taiwan’s recorded history struck the country. It led to 652 deaths, 47 missing people, and damage to property and infrastructure of over 3 billion USD^44^. In 5 days, typhoon Morakot brought over 2884 mm of precipitation to southern Taiwan, which caused severe floods and induced more than 22,705 landslides covering an area of 274 km^2^. The landslides were mainly characterized as shallow, but some deep-seated landslides also occurred across the mountainous regions of southern Taiwan^45^. For this study, we chose an area in the Western region of Taitung County in the south of the country as a case study area. Since this study’s objective was not to map all the landslides in southern Taiwan (Fig. 1a), we selected a region with an area of 467.91 km^2^ to train and test our model. Unlike the Eastern Iburi case study, there was no proper database on which to base a landslide inventory map for southern Taiwan. So, based on Google Earth’s archive images (2011–2013), we digitized every landslide within our selected area. In total, we digitized 895 landslides with a total area of 31.33 km^2^. The maximum, minimum, and mean of the landslide features are 1,455,022.72 m^2^, 1282.28 m^2^, and 34,359.85 m^2^ respectively.

#### Shuzheng Valley (China)

Shuzheng Valley is part of the Jiuzhaigou valley scenic and historic interest area located in Sichuan Province, China. The region is a popular tourist destination due to its diverse forest ecosystems, lakes, and landscapes^46^. On August 8, 2017, an earthquake with a magnitude (Mw) 7 struck Shuzheng Valley and the Jiuzhaigou scenic area, which resulted in 25 deaths and 525 people suffering from injuries, as well as the destruction of tourist resorts and an economic loss of 21 million USD^47^. A series of geohazards followed the earthquake, including a dam break and landslides, particularly in the Shuzheng Valley. The earthquake triggered 1780 mostly shallow landslides. However, due to the area’s rough topography, there were also rockfalls and rock avalanches with sizes ranging from a few cubic meters to thousands of cubic meters. For this case study, due to keeping the balance of the distribution of landslide features for training and testing in Shuzheng Valley, we selected a small region with an area of 64.4 km^2^ adjacent to this valley as our study area. The valley has a rough topography and steep slopes with elevations ranging between 1978 and 3711 m above MSL. Using Google Earth’s archive images (2018–2019), we digitized 212 landslide features with a total area of 2.74 km^2^ within the study area. The maximum, minimum, and mean of the landslide features are 130,641.49 m^2^, 1147.51 m^2^, and 11,571.6 m^2^, respectively (Fig. 1b).

### Sentinel-2 multispectral imagery

Google Earth Engine (GEE) environment is a cloud system developed by Google for processing satellite images. We used GEE to acquire cloud-free Sentinel-2images. Currently, GEE provides two Sentinel-2 products called Level-1C and Level-2A, whereby the former has global coverage but no atmospheric correction and the latter includes atmospheric correction but does not have global coverage. Therefore, we called and masked the Sentinel-2 Level-1C product for each study site and then imported it to Sen2Cor 50 plugin in SNAP software developed by the European Space Agency (ESA) to apply atmospheric corrections. We used the Sentinel-2A product rather than Sentinel-2B because it had less cloud cover for all three sites. The Copernicus Sentinel-2 mission is a constellation of two polar-orbiting satellites for earth observation. It uses Multispectral Instrument (MSI) sensors to acquire optical imagery. These images are acquire at various spatial resolutions ranging from 10 to 60 m, and 13 bands of visible, near-infrared and short-wave infrared electromagnet spectrum^48^. This constellation has two satellites, Sentinel-2A and 2B, at antipodal points in the same orbit, which provides a high revisit time of 5 days. The main themes of the Sentinel-2 mission are climate change, land monitoring, and emergency management, which includes mapping and monitoring landuse/landcover changes, forests, farmlands, water resources, and natural hazards. More information on the Sentinel-2 constellation is available in the User Handbook^49^. In this study, we only used the high-resolution image bands blue (2), green (3), red (4), and near-infrared (8) of Sentinel-2A with a 10-m spatial resolution to acquire imagery for the three study areas. Table 1 provides information on the acquired images for each site.

**Table 1:** Details of images acquired for each site.

Site	Acquisition date	Scene cloud cover (%)	Coverage area (ha)	Product	Pixel size (m)	Acquired bands
Japan	06.08.2019	2	345.64	Level-1C	10	2, 3, 4, 8
Taiwan	20.07.2016	4	467.91	Level-1C	10	2, 3, 4, 8
China	14.07.2018	4	64.4	Level-1C	10	2, 3, 4, 8

### Fully convolutional network (FCN)

One of the common ways in which DL approaches learn to deal with features with various shapes and sizes is by increasing the depth of the algorithm and using more convolutional layers. However, adding more layers usually causes a training degradation problem^35^. Long et al.^50^ introduced FCN, which does not have any fully connected (FC) layer, but instead replaces convolutional and upsampling layers to increase the training capability. The FCN is designed to represent image-to-image mapping, which is suitable for calculating per-pixel probability labels^51^. Therefore, the sample image patches with arbitrary sizes are introduced as the input for the algorithm^52^. Since there is no FC layer in an FCN, the algorithm can learn to recognize various representations of the local spatial input^53^. Moreover, FCNs also have skip connections between the down and up samplings for refining image segmentation^35,54^. Of the various types of FCN algorithms, the U-Net has received much interest. It is a simple and effective algorithm for feature extraction that can be trained by a limited number of sample patches^34,55^.

#### U-Net

U-Net was initially been introduced in the context of bio-medical image segmentation^51^ and further adopted in a variety of semantic segmentation tasks, generally achieving good performances^56^. The U-Net architecture consists of a contracting path (an encoder) to capture low-level representations, along with an expanding path (a decoder) to capture the high-level ones^34^. While the expanding path is an asymmetrical structure for retaking the vanished information of the feature localization^57^, the contracting path is similar to the standard CNN architectures, made up of consecutive convolution blocks. Each block contains two convolutional layers with a filter size of 3 × 3, leveraging a rectified linear unit (ReLU) activation function, and a max-pooling layer with a filter size of 2 × 2 and a stride of 2. After each convolution block, the number of feature maps is doubled, and a total of 512 feature maps are generated after the last block. The expanding path is an inverted form of the contracting one, whereby the input to a certain decoder block is represented by the concatenation of the previously outputted feature map and the corresponding output of the encoder block at the same level. The number of feature maps over the expanding path is halved after each block^51^. Overall, the U-Net algorithm implemented in our case comprise a total of 23 convolutional layers, including 19 convolutional and 4 convolutional-transpose ones. The U-Net structure is summarized in Table 2.

**Table 2:** Network structure of the U-Net architecture.

Module	Layer name	Kernel size	Stride	Kernel number	Output size
Input					128 × 128 × 4
	Conv 1	3 × 3	1	16	128 × 128 × 16
	Conv 2	3 × 3	1	16	128 × 128 × 16
	Max-pooling	2 × 2	2	16	64 × 64 × 16
	Conv 3	3 × 3	1	32	64 × 64 × 32
	Conv 4	3 × 3	1	32	64 × 64 × 32
	Max-pooling	2 × 2	2	32	32 × 32 × 32
	Conv 5	3 × 3	1	64	32 × 32 × 64
	Conv 6	3 × 3	1	64	32 × 32 × 64
Encoding	Max-pooling	2 × 2	2	64	16 × 16 × 64
	Conv 7	3 × 3	1	128	16 × 16 × 128
	Conv 8	3 × 3	1	128	16 × 16 × 128
	Max-pooling	2 × 2	2	128	8 × 8 × 128
	Conv 9	3 × 3	1	256	8 × 8 × 256
	Conv 10	3 × 3	1	256	8 × 8 × 256
	Conv-transpose	3 × 3	2	128	16 × 16 × 128
	Conv 11	3 × 3	1	128	16 × 16 × 128
Decoding	Conv 12	3 × 3	1	128	16 × 16 × 128
	Conv-transpose	3 × 3	2	64	32 × 32 × 64
	Conv 13	3 × 3	1	64	32 × 32 × 64
	Conv 14	3 × 3	1	64	32 × 32 × 64
	Conv-transpose	3 × 3	2	32	64 × 64 × 32
	Conv 15	3 × 3	1	32	64 × 64 × 32
	Conv 16	3 × 3	1	32	64 × 64 × 32
	Conv-transpose	3 × 3	2	16	128 × 128 × 16
	Conv 17	3 × 3	1	16	128 × 128 × 16
	Conv 18	3 × 3	1	16	128 × 128 × 16
Output	Conv 19	1 × 1	1	1	128 × 128 × 1

#### Residual U-Net

**Figure 2:**
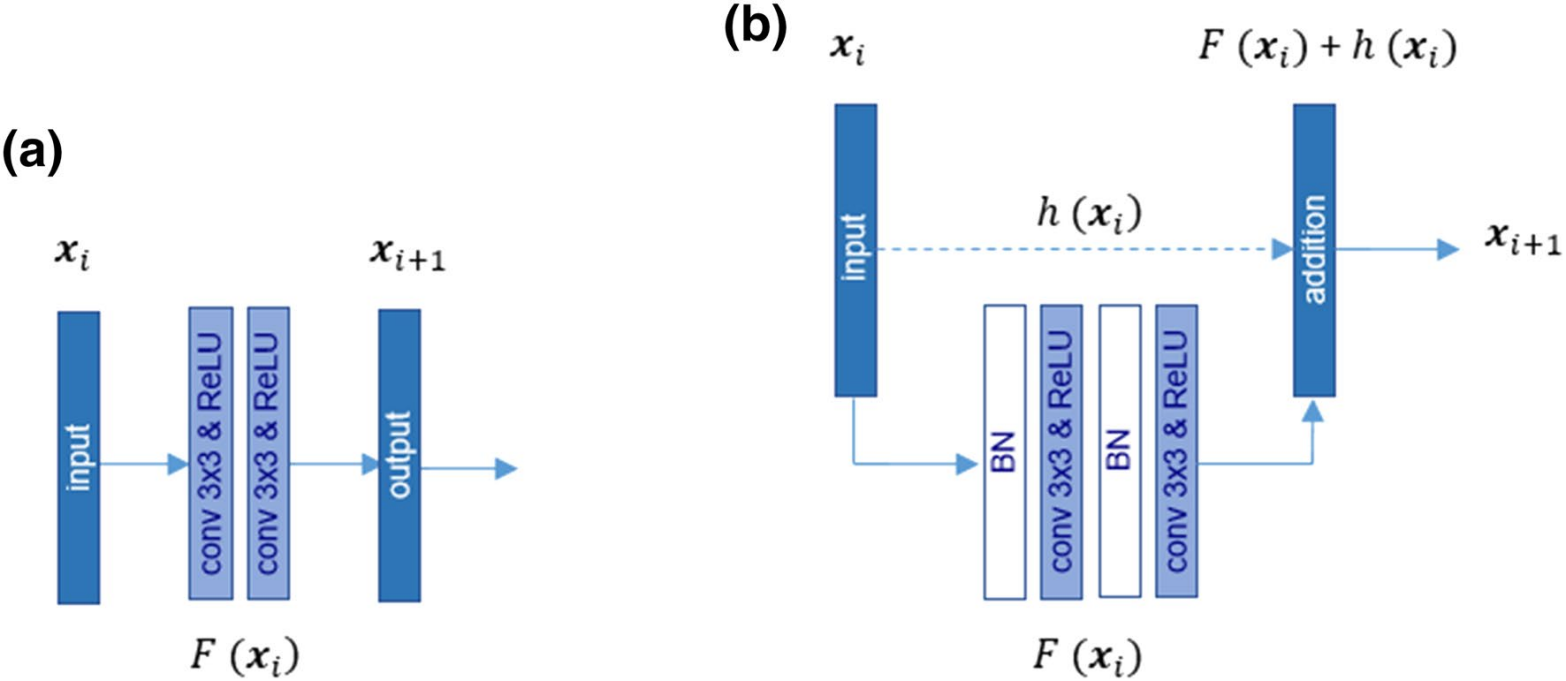
Illustration of the architecture of a typical convolutional network block in a U-Net (**a**) and a residual block with an identity mapping of $h \left({{\varvec{x}}}_{i}\right)$ (**b**).

The ResU-Net design is a variant of the U-Net algorithm, leveraging residual learning blocks. This modification is shown to often improve learning performance^58^, and can even avoid the vanishing gradient problems^59^. The architecture of a residual neural block is described as a stacked sequence of residual units, whereby a single residual unit is defined as:

1 $${{\varvec{y}}}_{i}=h \left({{\varvec{x}}}_{i}\right)+F ({{\varvec{x}}}_{i}, {W}_{i})$$

2 $${{\varvec{x}}}_{i+1}=f ({{\varvec{y}}}_{i})$$

whereby ${{\varvec{x}}}_{i}$ and ${{\varvec{x}}}_{i+1}$ refer to the input and output of the $i$ th residual unit; $f ({{\varvec{y}}}_{i})$ and $F (\cdot )$ are the activation and the residual functions, respectively; and $h (\cdot )$ is the identity mapping $h \left({{\varvec{x}}}_{i}\right)= {{\varvec{x}}}_{i}$. The convolutional layer reduces the spatial resolution of the applied sample patch image in the feature maps so that the dimension of the input (${{\varvec{x}}}_{i})$ might be higher than that of the output ($F \left({{\varvec{x}}}_{i}\right))$. Therefore, a linear projection ${W}_{i}$ is applied to maintain the dimension of the input and output of the convolutional layers (see Fig. 2).

**Figure 3:**
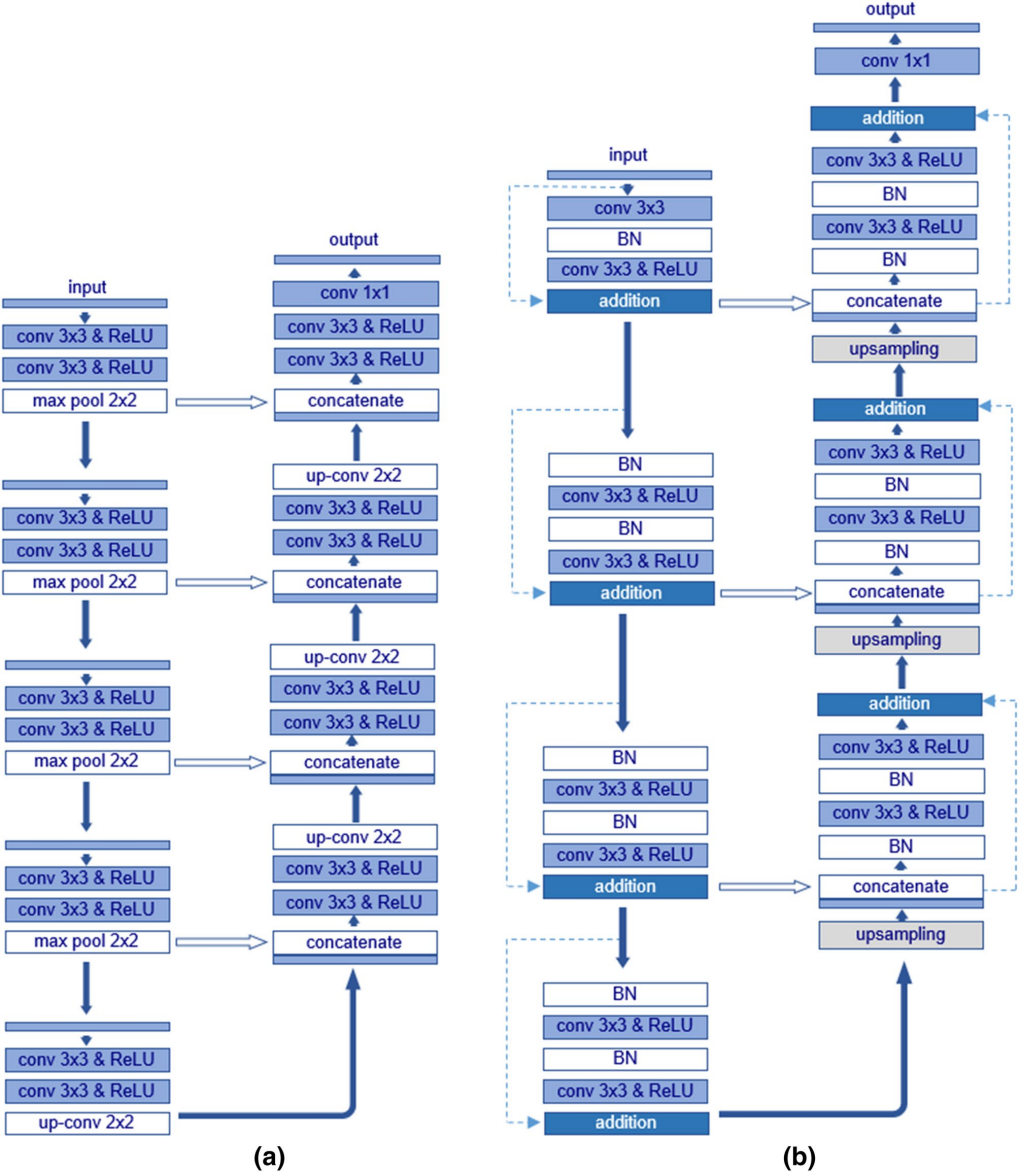
Architecture of the applied (**a**) U-Net and (**b**) ResU-Net in this study.

In the ResU-Net, the 2 × 2 max-pooling layer is absent, and the downsampling process is instead obtained with a convolution stride of 2. Moreover, a batch normalization (BN) procedure is inserted before each convolutional layer. Finally, the identity mapping $h \left({{\varvec{x}}}_{i}\right)$ adds the input of a block to its output. The expanding path comprises three residual learning blocks, each of which is preceded by a corresponding upsampling layer (Conv2DTranspose). To generate the statistical probabilities of the semantic categorization, a final convolutional layer with a 1 × 1 filter size and a sigmoid activation function are added on top of the ResU-Net architecture to associate each pixel to a corresponding output probability value comprised between 0 and 1. Thereby, the probability of a pixel belonging to each of the pre-defined segmentation categories is reported, which is relevant for solving our defined classification problem. The overall network structure of our applied ResU-Net consists of 15 convolutional layers, as listed in Table 3. To better depict a visual overview of the network architecture, Fig. 3 shows a schematic representation of the U-Net and ResU-Net structures.

**Table 3:** Network structure of the ResU-Net.

Module	Layer Name	Kernel Size	Stride	Kernel Number	Output Size	Output Size	Output Size
Input					128 × 128 × 4	64 × 64 × 4	32 × 32 × 4
	Conv 1	3 × 3	1	64	128 × 128 × 64	64 × 64 × 64	32 × 32 × 64
	Conv 2	3 × 3	1	64	128 × 128 × 64	64 × 64 × 64	32 × 32 × 64
Encoding	Conv 3	3 × 3	2	128	64 × 64 × 128	32 × 32 × 128	16 × 16 × 128
	Conv 4	3 × 3	1	128	64 × 64 × 128	32 × 32 × 128	16 × 16 × 128
	Conv 5	3 × 3	2	256	32 × 32 × 256	16 × 16 × 256	8 × 8 × 256
	Conv 6	3 × 3	1	256	32 × 32 × 256	16 × 16 × 256	8 × 8 × 256
	Conv 7	3 × 3	2	512	16 × 16 × 512	8 × 8 × 512	4 × 4 × 512
	Conv 8	3 × 3	1	512	16 × 16 × 512	8 × 8 × 512	4 × 4 × 512
	Conv 9	3 × 3	1	256	32 × 32 × 256	16 × 16 × 256	8 × 8 × 256
	Conv 10	3 × 3	1	256	32 × 32 × 256	16 × 16 × 256	8 × 8 × 256
Decoding	Conv 11	3 × 3	1	128	64 × 64 × 128	32 × 32 × 128	16 × 16 × 128
	Conv 12	3 × 3	1	128	64 × 64 × 128	32 × 32 × 128	16 × 16 × 128
	Conv 13	3 × 3	1	64	128 × 128 × 64	64 × 64 × 64	32 × 32 × 64
	Conv 14	3 × 3	1	64	128 × 128 × 64	64 × 64 × 64	32 × 32 × 64
Output	Conv 15	1 × 1	1	1	128 × 128 × 1	64 × 64 × 1	32 × 32 × 1

### Evaluation metrics

The resulting binary maps of areas detected as landslides were compared with the ground truth inventories in the holdout testing areas to calculate the precision, recall, and f1-score accuracy assessment metrics. The metrics were calculated based on true positives (TPs), which are the correctly detected landslide areas, false positives (FPs), which are the non-landslide areas that have been incorrectly detected as landslides, and false negatives (FNs), which are the landslide areas that have not been detected by the algorithm. The precision metric denotes the proportion of areas that were correctly identified as landslide areas. The recall, also known as the sensitivity metric, is the proportion of areas in the results that were identified as landslide areas. The f1-score is a quantitative metric that is useful to assess the balance between precision and recall (see Eqs. 3–5).

3 $$Precision=\frac{TP}{TP+FP}$$

4 $$Recall=\frac{TP}{TP+FN}$$

5 $$F1-score =2 \times \frac{Precision \times Recall}{Precision + Recall}$$

### Implementation details

**Figure 4:**
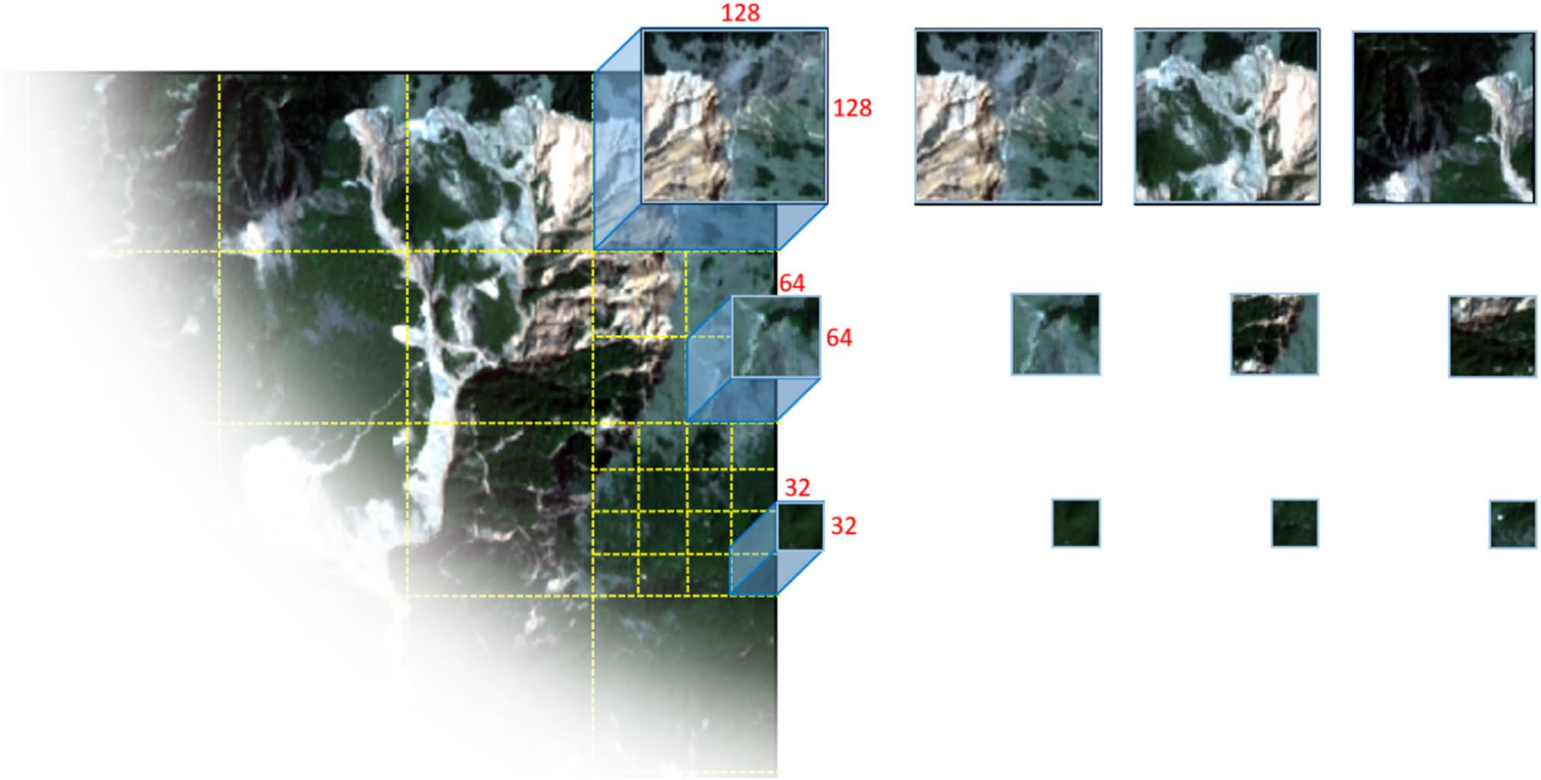
Systematic sample patch generation of input images by regular grid approach without any overlap. The maps were created using the ArcMap v.10.8 software (https://desktop.arcgis.com/es/arcmap/).

Each of the three images under examination (representing three different study areas) was divided into a training area and a testing area in a 3:2 ratio. This study is the first attempt to use only 10-m resolution satellite imagery for landslide detection using FCNs and so far there is no optimal modified sample patch size for this purpose. Therefore, three different window sizes (32 × 32, 64 × 64, and 128 × 128 pixels) were used to generate the input sample patches by applying a regular grid approach without any overlap or data augmentation. In total, 7888, 1972, and 592 sample patches were generated based on the window sizes of 32 × 32, 64 × 64, and 128 × 128 pixels, respectively (see Fig. 4).

To assess the transferability of the models and the impact of different areas on the network performance, the following scenarios were defined, and each of them was evaluated on the testing areas of Taiwan, China, and Japan:

- Scenario 1: training the models on the collection of the three training sets.
- Scenario 2: training the models on the Taiwan training section only.
- Scenario 3: training the models on the China training section only.
- Scenario 4: training the models on the Japan training section only.

**Figure 5:**
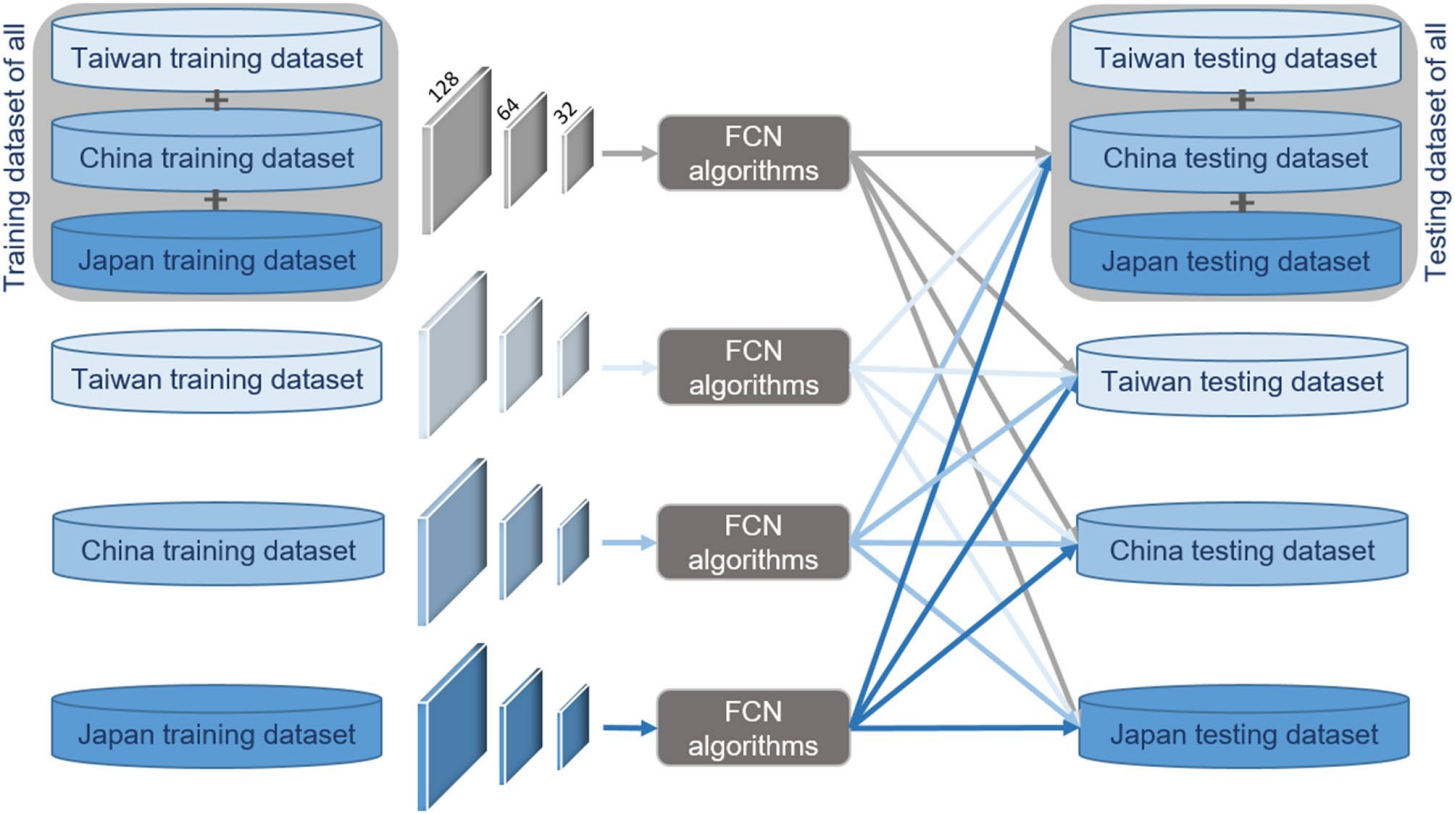
The scheme summarizing the four different scenarios in this study.

Moreover, the four scenarios were tested for each of the three different window sizes; therefore, each testing area was described by a total of 16 different results (see Fig. 5).

We used the binary cross-entropy for both U-Net and ResU-Net. The cross-entropy was used as the loss function to find the difference between each ${Pl (x)}^{(x)}$ from the highest probability of 1 using Eq. (6).

6 $$E= \sum_{x=1}^{K}w \left(x\right){\mathrm{log}}_{\mathit{Pl} (x)}(x)$$

where $K$ is the number of classes and $w$ is a weight map, which is introduced as the pixels that were more important than the others in the training process^51^. The models were trained by backpropagation through mini-batch stochastic training and the Adam optimization algorithm^60^, setting a learning rate of 0.001 (with *β*_*1*_ = 0.9, and *β*_*2*_ = 0.999). The batch size was chosen to include four images per step; the optimal results were derived by following an early stopping approach based on the evaluation loss.

## Results and accuracy assessment

We used the same input data to train and test both algorithms in different scenarios to compare their performance and transferability. The algorithms were evaluated on the defined testing areas using the precision, recall, and f1-score accuracy assessment metrics.

### Scenario 1 and accuracy

**Figure 6:**
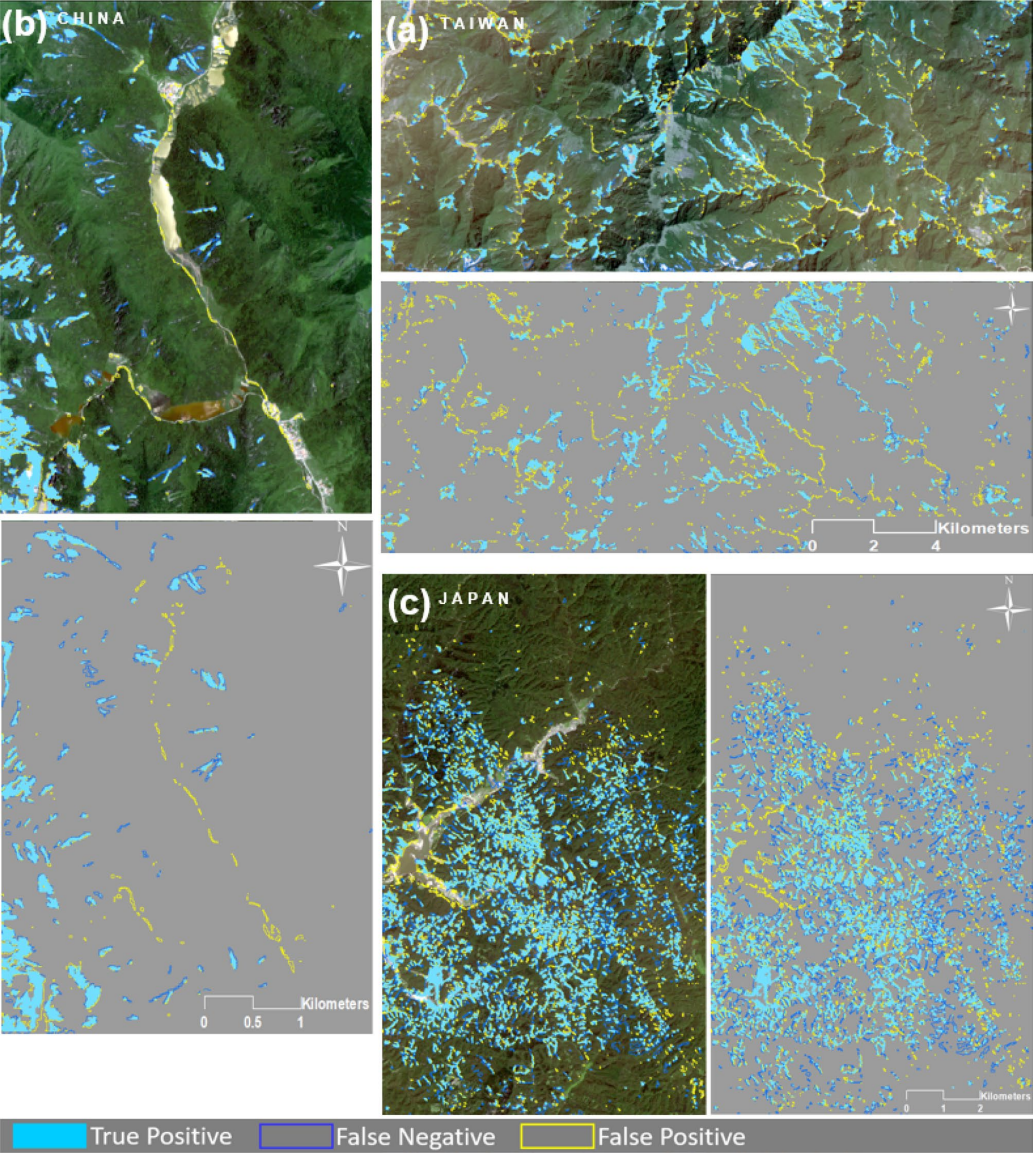
Results of the ResU-Net trained by all training datasets using a sample patch size of 64 × 64 pixels. The maps were created using Python 3.6 (https://www.python.org/) and the ArcMap v.10.8 software (https://desktop.arcgis.com/es/arcmap/).

In this scenario, both U-Net and the ResU-Net algorithms were trained with a dataset containing sample patches from all training areas of Taiwan, China, and Japan. The testing procedure was carried out based on the dataset with sample patches from holdout testing areas of each study area and with data from all our study areas. The algorithms were trained and tested separately based on sample patch window sizes of 32 × 32, 64 × 64, and 128 × 128 pixels. For simplicity, Fig. 6 only shows the results of the ResU-Net trained by all training datasets using a sample patch size of 64 × 64 pixels. The accuracy assessment metrics were calculated for each resulting landslide detection map (see Table 4). ResU-Net obtained the higher f1-score values of just under 73% and 71.29% tested on Taiwan's testing area using a sample patch size of 64 × 64 pixels and 32 × 32 pixels, respectively. Testing the ResU-Net on the Taiwan test area with the sample patch size of 128 × 128 pixels achieved the lowest f1-score. Similarly, testing the U-Net on the Taiwan test area with the same sample patch size of 128 × 128 also achieved the lowest f1-score value of 68.48%. The highest recall value was achieved in the Taiwan case study area with 88.38% for the rest-Net with a sample patch size of 128 × 128 pixels, while the highest recall value of 84.16% using the U-Net was achieved with a sample patch size of 64 × 64. Although the trained algorithms with all training datasets achieved the highest f1-score and recall values in the case study area of Taiwan, the highest precision value of 81.2% was achieved by testing the U-Net on the China testing area with a sample patch size of 128 × 128 pixels. Higher recall values were generally obtained by testing the algorithms on the case study area of Taiwan, and higher precision values were achieved in that of China. This means that when algorithms were trained by all training datasets, they were able to detect most of the landslide areas in China and yield fewer incorrectly identified landslide areas than in Taiwan.

**Table 4:** The resulting precision, recall, and f1-score values for Scenario 1 (training the algorithms with all training datasets and testing them on the Taiwan, China, Japan, and all test datasets separately).

		U-Net			ResU-Net	
Size	32	64	128	32	64	128
Precision	67.45	63.95	64.32	66.39	69.65	60.11
Recall	58.97	67.13	61.39	6543	63.85	74.29
F1-score	62.93	65.5	62.82	65.91	66.63	66.45
Precision	65.47	60.48	59.73	64.99	68.11	57.21
Recall	77.52	84.16	80.24	78.94	78.51	**88.38**
F1-score	70.99	70.38	68.48	71.29	**72.94**	69.46
Precision	79.76	70.27	**81.2**	75.02	74.74	65.57
Recall	55.52	61.85	55.48	59.04	60.33	64.3
F1-score	65.47	65.79	65.92	66.07	66.76	64.93
Precision	69.26	68.35	70.8	67.33	71.12	63.41
Recall	44.05	53.62	46.33	54.92	52.14	63.43
F1-score	53.85	60.1	56.01	60.49	60.17	63.42
Algorithms trained by sample from			All	Taiwan	China	Japan

The highest values of precision, recall, and f1-score are indicated in bold.

### Scenario 2 and accuracy

**Figure 7:**
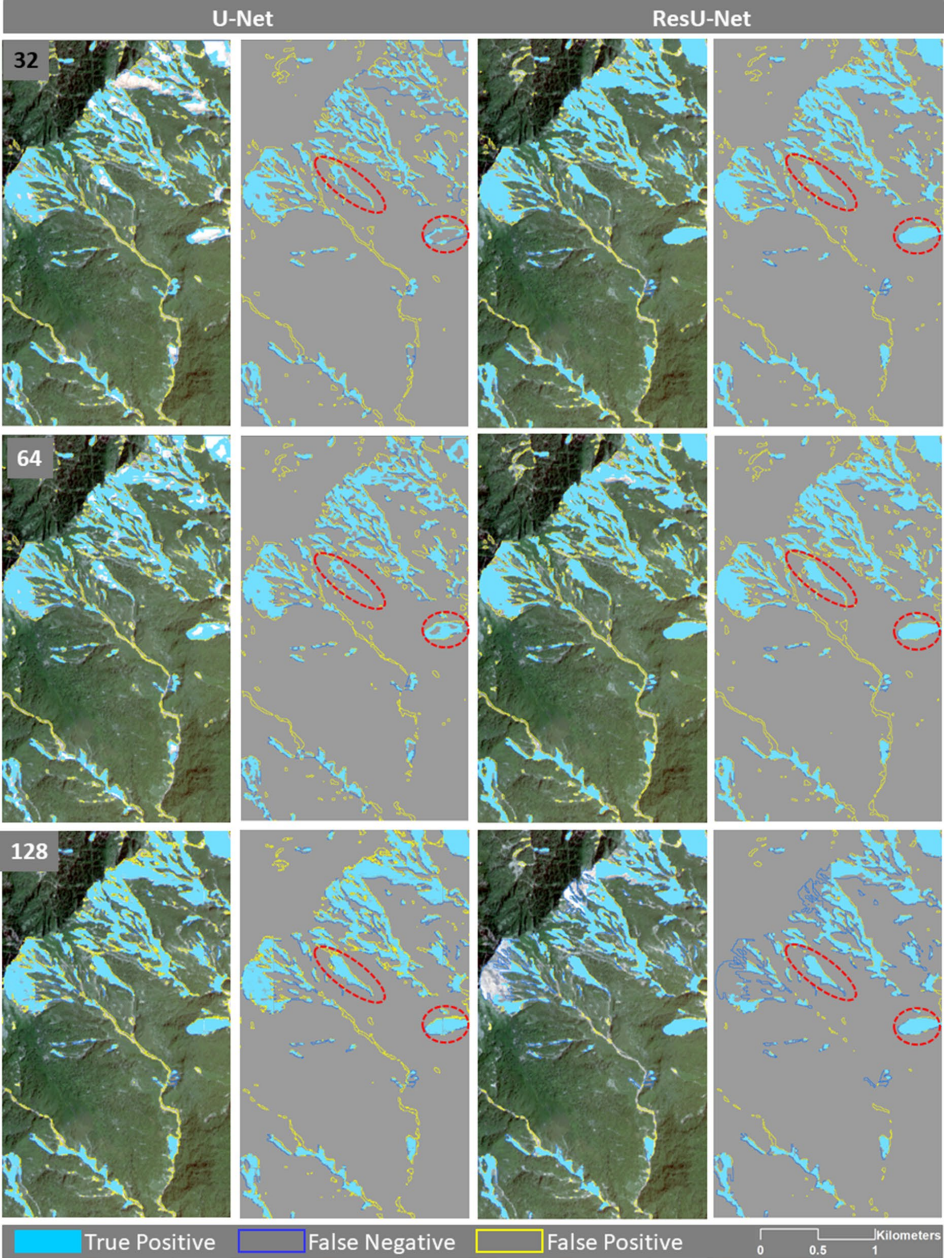
The enlarged areas of the results of the U-Net and ResU-Net trained and tested on Taiwan datasets using different sample patch sizes. Red ovals are showing examples of differences in the results. The maps were created using Python 3.6 (https://www.python.org/) and the ArcMap v.10.8 software (https://desktop.arcgis.com/es/arcmap/).

To evaluate the generalisation performance of the algorithms, they were also trained separately with data from each individual training area of each case study. Figure 7 shows the results in only an enlarged area from the Taiwan testing area. According to this figure, both algorithms that trained and tested on Taiwan datasets represent most of the false positives within the runouts.

The accuracy of the algorithms that were trained only with the training dataset of the case study area of Taiwan was also evaluated based on Taiwan, China, Japan, and all test datasets separately and the resulting values are given in Table 5. ResU-Net again obtained the higher f1-score values of 72.68%, and 70.54% tested on Taiwan's testing area using a sample patch size of 64 × 64 pixels and 32 × 32 pixels, respectively. This algorithm also resulted in the highest recall value of 80.72% in the same study area using a sample patch size of 32 × 32 pixels followed by 79.65% achieved with a sample patch size of 128 × 128 pixels using the U-Net. Therefore, training the algorithms with the Taiwan dataset and testing the algorithm against its test area was helpful in these two sample patch sizes to significantly reduce incorrect identification of non-landslide areas as landslides. However, its ability to correctly detect landslide areas was significantly higher in Japan using the sample patch sizes of 128 × 128 pixels, which achieved a precision value of well over 82%.

**Table 5:** The resulting precision, recall, and f1-score values for Scenario 2 (training the algorithms with the training dataset of Taiwan and testing it on the Taiwan, China, Japan, and all test datasets separately).

		U-Net			ResU-Net	
Size	32	64	128	32	64	128
Precision	63.12	60.22	62.7	62.64	70.21	68.06
Recall	42.46	45.04	48.9	40.73	45.06	35.64
F1-score	50.77	51.53	54.95	49.36	54.89	46.78
Precision	67.45	54.47	57.81	61.7	68.75	61.29
Recall	62.92	70.48	79.65	**80.72**	77.08	77.3
F1-score	63.68	61.45	67	70.54	**72.68**	68.37
Precision	78.41	69.62	75.8	66.31	69.55	79.19
Recall	53.61	50.92	48.65	43.53	44.33	44.87
F1-score	63.68	58.82	59.26	52.56	54.15	57.29
Precision	78.49	78.41	79.21	73.55	75.78	**82.31**
Recall	24.63	23.6	23.66	21.19	18.82	14.65
F1-score	37.5	36.28	36.43	32.9	30.16	24.87
Algorithms trained by sample from			All	Taiwan	China	Japan

The highest values of precision, recall, and f1-score are indicated in bold.

### Scenario 3 and accuracy

**Figure 8:**
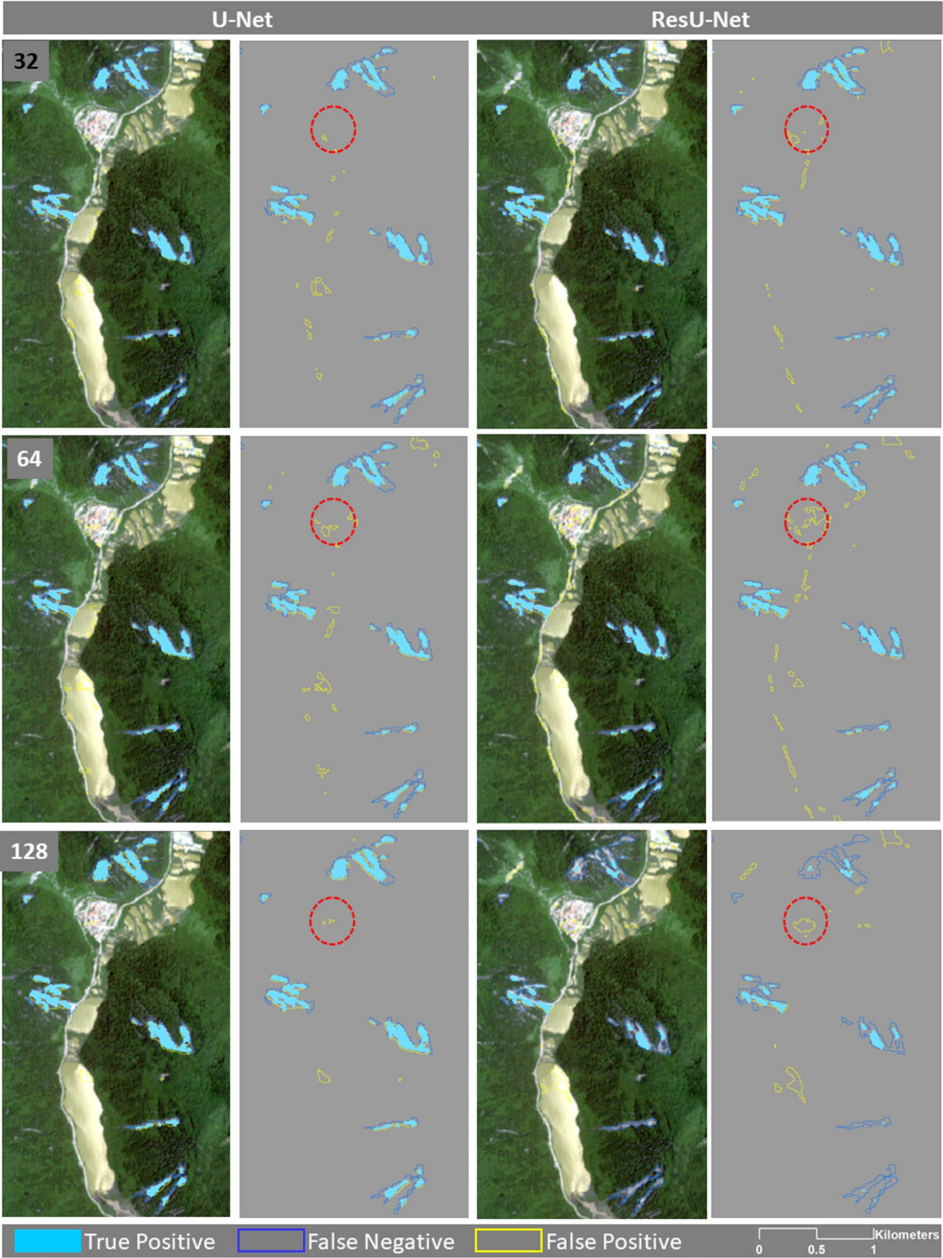
The enlarged areas of the results of the U-Net and ResU-Net trained and tested on China datasets using different sample patch sizes. Red ovals are showing examples of differences in the results. The maps were created using Python 3.6 (https://www.python.org/) and the ArcMap v.10.8 software (https://desktop.arcgis.com/es/arcmap/).

In this scenario, both U-Net and the ResU-Net algorithms were trained with the China training dataset (see Fig. 8), where the ResU-Net algorithm was again able to achieve the highest f1-score value of 72.9% (see Table 6). The accuracy assessment metric values of this scenario fluctuated around 69% for the U-Net algorithm with three different sample patch sizes, and the highest one was over 70% for a window size of 32 × 32 pixels. The U-Net algorithm also achieved the highest recall value of approximately 91% but in Taiwan's holdout testing area. The ResU-Net also showed a substantially good recall value of 89.92% in Taiwan. The highest recall value of the U-Net algorithm was achieved with a 64 × 64 pixel sample patch size, whereas the highest recall value of the ResU-Net algorithm was achieved with a 128 × 128 pixel sample patch size. Like in the previous scenario, high precision values were achieved with both algorithms in Japan’s case study area. However, the highest precision value of 81.25 was achieved with U-Net with a window size of 128 × 128 pixels. However, the resulting recall values were lower compared to those of the other study areas, which means that although the algorithms could detect many of the landslide areas in Japan, at the same time, they mistakenly detected several non-landslide areas that displayed similar spectral and slope information as the landslides, but whose characteristics were not represented in the Chinese training dataset.

**Table 6:** The resulting precision, recall, and f1-score values for Scenario 3 (training the algorithms with the training dataset of China and testing on the Taiwan, China, Japan, and all test datasets separately).

		U-Net			ResU-Net	
Size	32	64	128	32	64	128
Precision	58.61	46.68	51.98	47.19	43.33	42.42
Recall	56.57	60.75	59.69	61.75	53.35	59.37
F1-score	57.57	52.8	55.57	53.5	47.85	49.48
Precision	50.31	37.95	43.17	35.95	29.36	34.09
Recall	81.25	**90.9**	87.62	75.31	57.31	89.92
F1-score	62.14	53.55	57.84	48.67	38.83	49.44
Precision	78.59	75.52	**81.25**	72.25	75.91	71.74
Recall	63.55	64.66	59.58	71.09	69.92	65.4
F1-score	70.28	69.67	68.75	71.7	**72.9**	68.48
Precision	79.98	80.32	81.03	71.6	7312	**81.17**
Recall	35.66	35.64	36.76	50.05	48.46	33.84
F1-score	49.33	49.37	50.58	58.92	58.29	47.76
Algorithms trained by sample from			All	Taiwan	China	Japan

The highest values of precision, recall, and f1-score are indicated in bold.

### Scenario 4 and accuracy

**Figure 9:**
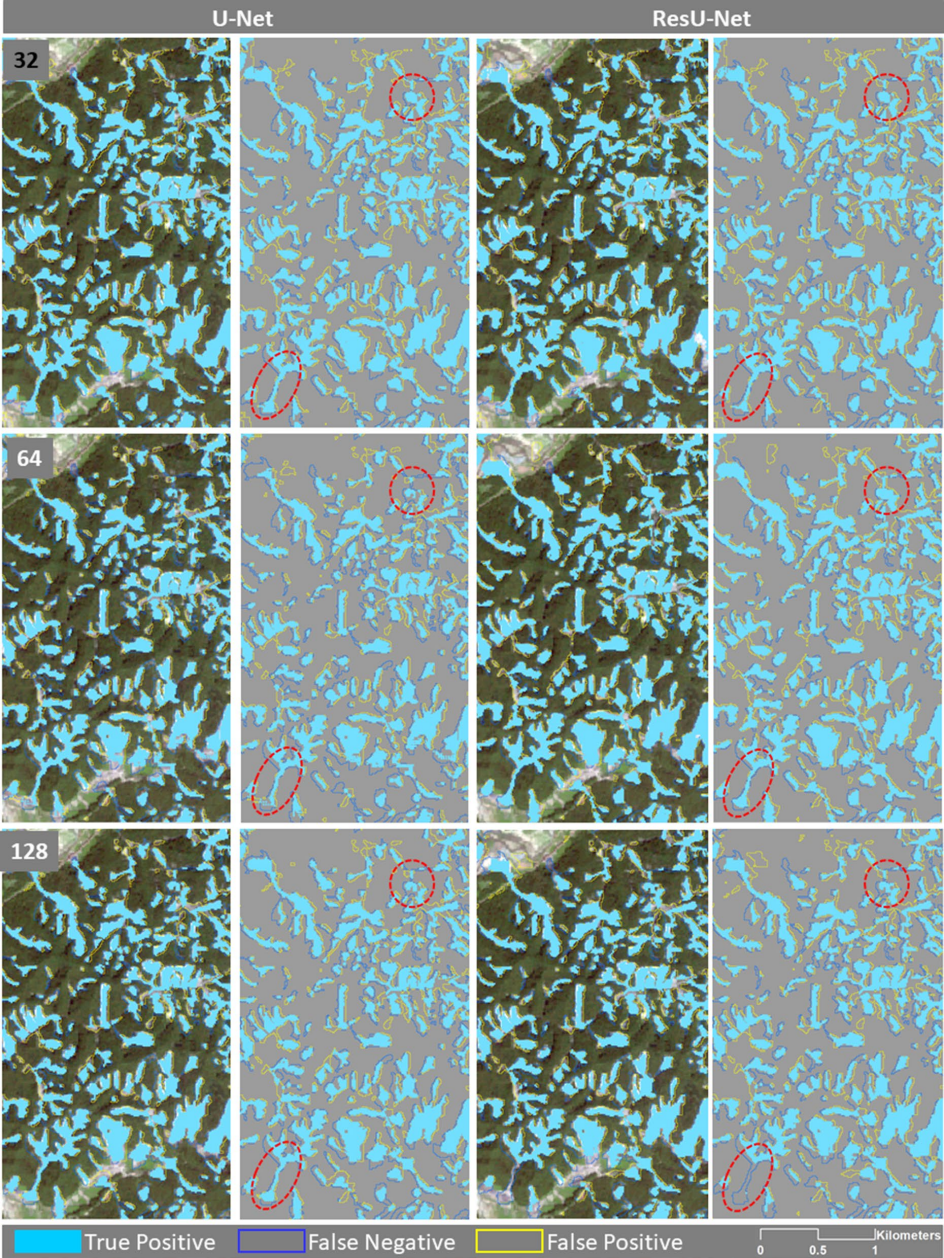
The enlarged areas of the results of the U-Net and ResU-Net trained and tested on the Japan datasets using different sample patch sizes. Red ovals showing examples of differences in the results. The maps were created using Python 3.6 (https://www.python.org/) and the ArcMap v.10.8 software (https://desktop.arcgis.com/es/arcmap/).

Although the last scenario, namely training the algorithms using the Japan dataset, showed a fairly good accuracy of the results for Japan itself (see Fig. 9 and Table 7), the highest accuracies achieved overall were those tested on the China dataset. The highest precision (71.82%) and f1-score (73.32%) values were obtained by ResU-Net using a sample patch size of 64 × 64 pixels. The highest recall values were achieved with U-Net in China’s case study area and were between 81.18 and 78.84% based on widow sizes of 32 × 32 and 64 × 64 pixels, respectively. The f1-score values obtained based on China’s holdout testing area fluctuated around 70%, whereas those of Japan were around 60%. Moreover, this scenario was not successful in testing Taiwan’s case study area as this dataset achieved the lowest f1-score, precision, and recall values of 31.2%, 27.07%, and 24.7%, respectively.

**Table 7:** The resulting precision, recall, and f1-score values for Scenario 4 (training the algorithms with the training dataset of Japan and testing on the Taiwan, China, Japan, and all test datasets separately).

		U-Net			ResU-Net	
Size	32	64	128	32	64	128
Precision	44.13	32.01	48.56	42.16	61.03	41.14
Recall	56.46	66.64	56.97	57.31	47.01	63.58
F1-score	49.54	43.25	52.43	48.58	53.11	49.95
Precision	28.75	21.72	35.17	27.07	42.34	29.58
Recall	51.46	80.87	59.38	52.51	24.7	73.95
F1-score	36.89	34.24	44.17	35.73	31.2	42.26
Precision	63.52	63.46	67.96	67.47	**71.82**	62.95
Recall	**80.18**	78.3	74.18	73.55	74.88	73.55
F1-score	70.89	70.1	70.93	70.38	**73.32**	67.84
Precision	67.58	66.44	70.89	66.53	69.85	68.91
Recall	58.51	53.89	53.85	59.36	62.79	54.35
F1-score	62.72	59.51	61.21	62.74	66.13	60.77
Algorithms trained by sample from			All	Taiwan	China	Japan

The highest values of precision, recall, and f1-score are indicated in bold.

## Discussion

### Transferability assessment

**Figure 10:**
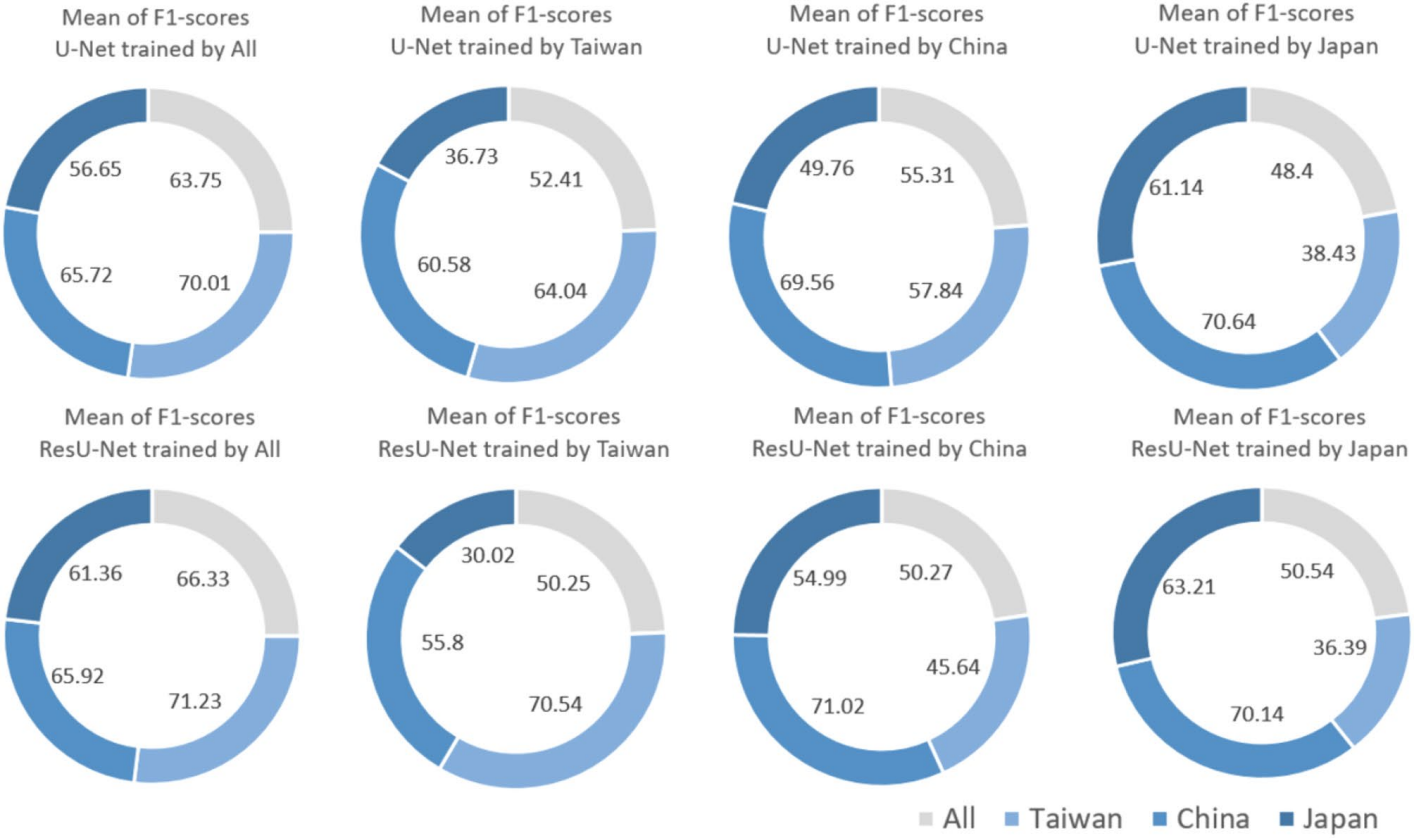
Overall mean F1-score values for U-Net and ResU-Net in relation to each of the four original scenarios.

We comprehensively assessed the performance and transferability of the algorithms by comparing the landslide detection of all the different possible combinations of training and testing areas. Therefore, while the first scenario is based on the collective training based on all the case study areas, the other scenarios singularly focus the training process on a certain specific area while still evaluating it on all three different study areas. Figure 10 shows the overall mean F1-score values for U-Net and ResU-Net in each of the four scenarios. The ResU-Net resulting scores were higher than the U-Net ones whenever the training and testing set belonged to the same study area. Specifically, ResU-Net achieves a mean f1-score value of 66.33 while U-Net achieves a value of 63.75 when training and testing based on all the study areas, and 70.54 versus 64.04 when training and testing in Taiwan, 71.02 versus 69.56 when training and testing in China, and 63.21 versus 61.14 when training and testing in Japan. A few curious findings are worth mentioning. The first scenario (collective training) leads to the best evaluation results for the Taiwan area, compared to the other testing areas, which can be due to probable higher similarities among the training areas of Japan and China and the test area of Taiwan. Furthermore, when training the models on the Japan training area, we observed that the highest mean f1-score values were obtained for China's testing area, and not Japan’s one (70.64 and 70.14 versus 61.14 and 63.21, for U-Net and ResU-Net, respectively). Finally, regarding the second and third scenarios (training with the Taiwan and China datasets, respectively), the highest mean f1-score was obtained for the local testing areas (namely Taiwan and China, respectively).

### Impact of sample input sizes on the network results

**Figure 11:**
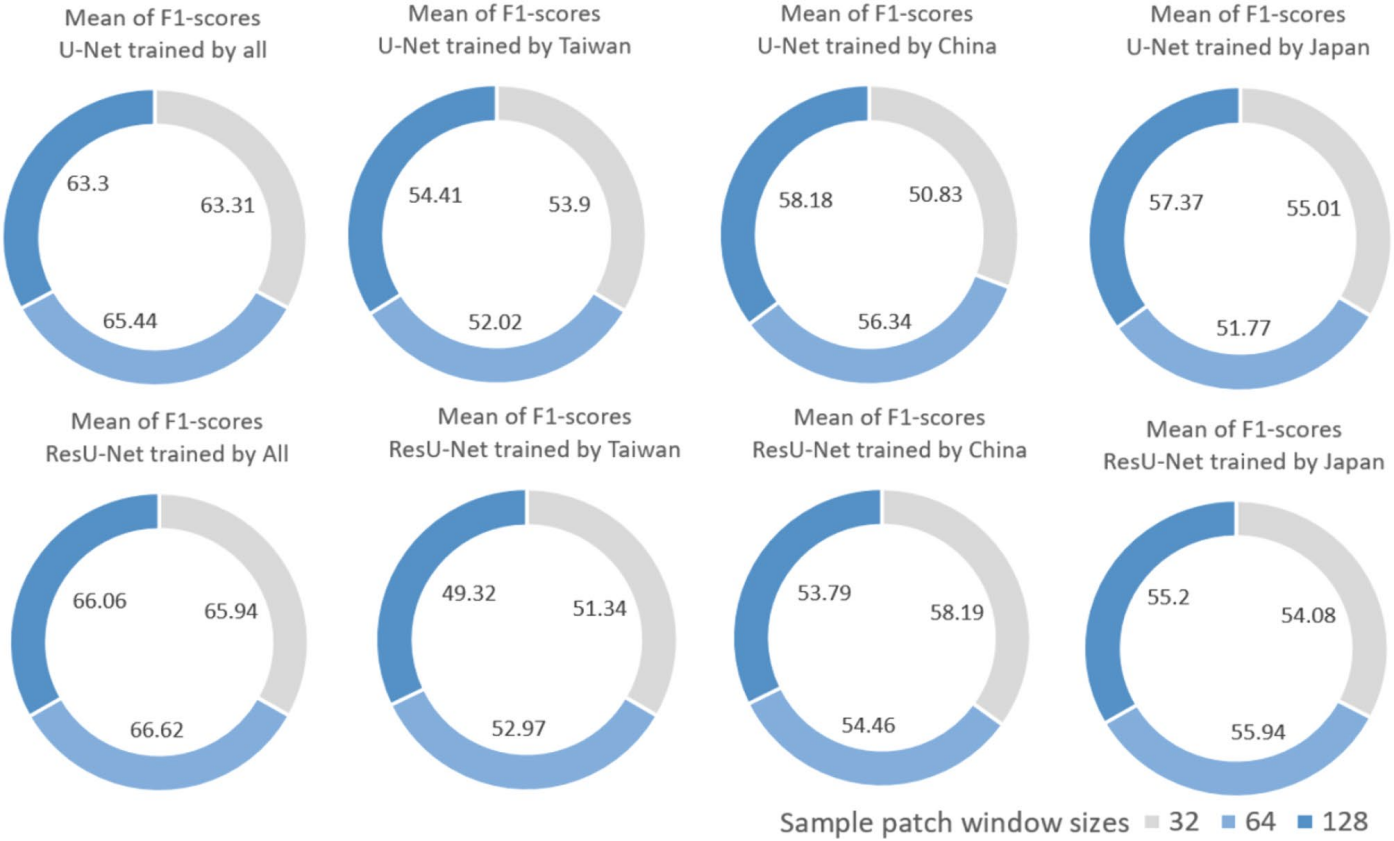
Comparison of the f1-score mean values for different sample patch window sizes (32 × 32, 64 × 64, and 128 × 128 pixels) in every scenario.

We sampled the images using a regular grid approach to evaluate three different window sizes, namely 32 × 32, 64 × 64, and 128 × 128 pixels. The resulting mean f1-score values of the U-Net model yielded a better performance for the sample size of 128 × 128 pixels, except for the first scenario. On the other hand, the ResU-Net achieved the best results with a sample size of 64 × 64 pixels, except for the third scenario. However, aside from a slightly superior performance when using 128 × 128 pixels for U-Net, and 64 × 64 pixels for ResU-Net, we did not observe a definitive advantage to guide the choice of window sizes that would potentially provide substantial improvements (see Fig. 11).

**Figure 12:**
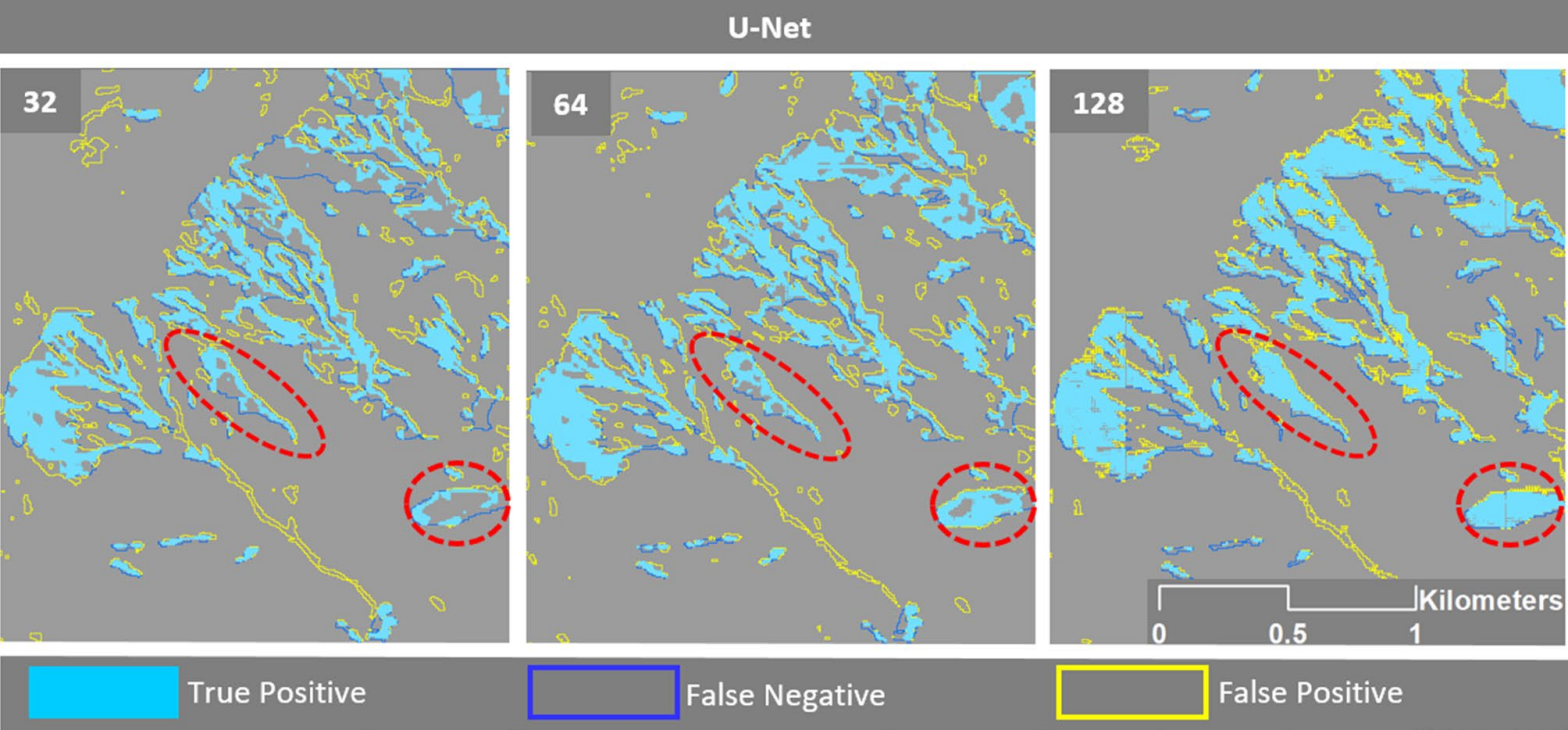
Comparison of how different window sizes affected the U-Net results in Taiwan. The maps were created using Python 3.6 (https://www.python.org/) and the ArcMap v.10.8 software (https://desktop.arcgis.com/es/arcmap/).

Whereas there is no clear evidence of a consistent impact of different window sizes in the multitude of assessed scenarios, Fig. 12 provides a visual example of the testing results of the U-Net model trained on the Taiwan dataset. The figure shows that, in this case, an increased window size led to higher recall values due to an increase in the correct detection of landslides (true positives), but not all cases followed this trend because of smaller landslide features.

### Challenges from imbalanced datasets

Since a total of 7888, 1972, and 592 sample patches were generated based on the window sizes of 32 × 32, 64 × 64, and 128 × 128 pixels, respectively, the extent of the images of the three study areas was very different, with Taiwan being the largest one with 467.91 ha. This leads us to conclude that the first scenario, where the models were trained on the combination of all datasets, performed better in Taiwan because most of the training data were based in Taiwan. The fourth scenario is a curious case, where both U-Net and ResU-Net, trained on the Japan training set, demonstrated better results when tested in China than in the testing area of Japan. We think that the spectral and textural features of the Japan training area are more similar to those of the China testing area than to the ones of the Japan testing area. This qualitatively triggers the hypothesis of a substantial heterogeneous profile of Japan’s landslide sample and a more homogeneous profile in China’s image, which is partially covered by a similar profile in a section of the Japan training area. It is indeed visible a high number of small landslide feature profiles in Japan that were not represented in the case study of China, While the bigger ones are present in both case study areas of China and Japan. Even if we selected study areas with the same area, the ratio between landslide areas and non-landslide areas might vary, as might the frequency and number of landslides in different geo-environmental case studies.

### Compared with recent works

The main objective of this study was to evaluate the performance of the U-Net and ResU-Net algorithms for landslide detection using freely available Sentinel-2 data and an ALOS DEM. Although, to our knowledge, our study is the first application of the U-Net and ResU-Net algorithms for landslide detection from freely available data, we compared our accuracy assessment results to those of the few recently published articles that applied these algorithms for landslide detection using VHR data. For instance, in the studies that were carried out by Liu et al*.*^34^, Qi et al.^35^, and Yi and Zhang^61^, the U-Net and ResU-Net algorithms were compared to each other in terms of their applicability for landslide detection from VHR imageries. Our results obtained more accurate landslide detection results using the ResU-Net than with the U-Net algorithm. In another instance, Soares et al.^33^ used only U-Net to evaluate different sample patch generation methods and window sizes for landslide detection from VHR satellite imagery. The same sample patch window sizes of 32 × 32, 64 × 64, and 128 × 128 pixels were used in their study, and the U-Net that was trained with 128 × 128 pixels achieved the highest f1-scores of 0.55 and 0.58 in two different testing areas. Our results confirmed theirs, as our U-Net yielded the highest mean f1-score values when trained with 128 × 128 pixels sample patches.

### Limitations

The application of U-Net and ResU-Net for landslide detection is associated with some issues. For instance, using these algorithms and image resolution could easily detect the big landslides from dense vegetation, but cases of neighboring bare land increased the false positives substantially. Nevertheless, the inclusion of topographic slope data helped to discriminate landslides from bare land in many cases. However, a precise and detailed extraction of landslides from bare land requires proper auxiliary data like displacement information from SAR data or prior expert knowledge, which was not used in this study. Further factors that need to be carefully considered in the future are the imbalanced nature of the dataset and a detailed analysis of the impact of the dataset size, which will help tackle the remaining unsolved issues.

Moreover, our initial expectation was that the global generalised performance would be improved by collectively merging training data from multiple geo-environmental case study areas. However, our hypothesis was not confirmed, and local training data based only on each target study area often outperformed a collective training dataset. Furthermore, it is not yet clear why a specific sample patch size yields better performance than another one in some specific local contexts and scenarios, and not in others, with fluctuations not directly following a consistent trend. This illustrates issues of low transparency related to the use of the proposed models.

## Conclusions and outlook

This work evaluated the generalization and transferability of two well-known FCN algorithms (U-Net and ResU-Net) for landslide detection in different scenarios. We demonstrated the effectiveness of these algorithms on landslide detection using freely available Sentinel-2 data and an ALOS DEM. We selected three different geo-environmental study areas in Taiwan, China, and Japan to train and test the algorithms. The applied semantic segmentation models were trained based on each individual area and on a combined dataset of all areas to detect landslides based on Sentinel-2 data and an ALOS DEM. To the best of our knowledge, no study has yet explored the possibility of using freely available satellite imagery for landslide annotation using FCN deep learning algorithms. Three different sample patch sizes were generated from pre-defined window sizes for training and testing the algorithms. Therefore, multiple experiments have been designed to evaluate the transferability of the algorithms and the impact of window sizes on different operational scenarios. Based on our results, we explored relationships among the applied models, the window sizes of sample patches, and the training datasets for landslide detection. Our results show that although the ResU-Net led to higher performances, the U-Net has more transferability capabilities. ResU-Net demonstrated the highest score in those cases where it was trained on only the local training datasets.

In future work, we aim to integrate the FCN algorithms with some frameworks that enable us to incorporate prior knowledge to different sections of FCNs, e.g., optimally selecting sample patch window size and location and enhancing the detection result by considering possible post-processing classification approaches.

## References

[1] Guzzetti F (2012). Landslide inventory maps: new tools for an old problem. Earth Sci. Rev..

[2] Bell R (2020). Major geomorphic events and natural hazards during monsoonal precipitation 2018 in the Kali Gandaki Valley. Nepal Himalaya. Geomo.

[3] Gariano SL, Guzzetti F (2016). Landslides in a changing climate. Earth Sci. Rev..

[4] Sun W (2017). Loess landslide inventory map based on GF-1 satellite imagery. Remote Sens. Basel.

[5] Ye C (2019). Landslide detection of hyperspectral remote sensing data based on deep learning with constrains. IEEE J. Sel. Top. Appl. Earth Obs. Remote Sens..

[6] Cui P, Zhu Y-Y, Han Y-S, Chen X-Q, Zhuang J-Q (2009). The 12 May Wenchuan earthquake-induced landslide lakes: distribution and preliminary risk evaluation. Landslides.

[7] Plank S, Twele A, Martinis S (2016). Landslide mapping in vegetated areas using change detection based on optical and polarimetric sar data. Remote Sens. Basel.

[8] Ghorbanzadeh O (2019). Evaluation of different machine learning methods and deep-learning convolutional neural networks for landslide detection. Remote Sens. Basel.

[9] Lima, P. *et al.* in *Workshop on World Landslide Forum.* 943–951 (Springer).

[10] Thai Pham B (2019). Landslide susceptibility assessment by novel hybrid machine learning algorithms. Sustainability.

[11] Wang H, Zhang L, Yin K, Luo H, Li J (2020). Landslide identification using machine learning. Geosci. Front..

[12] Xu, Q., Ouyang, C., Jiang, T., Fan, X. & Cheng, D. DFPENet-geology: a deep learning framework for high precision recognition and segmentation of co-seismic landslides. arXiv preprint https://arxiv.org/abs/1908.10907 (2019).

[13] Lissak C (2020). Remote sensing for assessing landslides and associated hazards. SGeo.

[14] Ghorbanzadeh O (2020). An application of Sentinel-1, Sentinel-2, and GNSS data for landslide susceptibility mapping. ISPRS Int. J. Geo Inf..

[15] Siyahghalati S, Saraf AK, Pradhan B, Jebur MN, Tehrany MS (2016). Rule-based semi-automated approach for the detection of landslides induced by 18 September 2011 Sikkim, Himalaya, earthquake using IRS LISS3 satellite images. Geomat. Nat. Haz. Risk.

[16] Mondini AC (2021). Landslide failures detection and mapping using Synthetic Aperture Radar: Past, present and future. Earth Sci. Rev..

[17] Doshida, S. *Workshop on World Landslide Forum.* 283–287 (Springer).

[18] Ghorbanzadeh O, Meena SR, Blaschke T, Aryal J (2019). UAV-Based Slope Failure Detection Using Deep-Learning Convolutional Neural Networks. Remote Sens. Basel.

[19] Tavakkoli Piralilou S (2019). Landslide detection using multi-scale image segmentation and different machine learning models in the higher himalayas. Remote Sens. Basel.

[20] Shi, W. *et al.* Landslide recognition by deep convolutional neural network and change detection. *ITGRS* (2020).

[21] Shahabi H (2019). A semi-automated object-based gully networks detection using different machine learning models: a case study of Bowen catchment, Queensland, Australia. Sensors.

[22] Blaschke T (2014). Geographic object-based image analysis–towards a new paradigm. ISPRS J. Photogramm. Remote. Sens..

[23] Blaschke T (2010). Object based image analysis for remote sensing. ISPRS J. Photogramm. Remote. Sens..

[24] Van Den Eeckhaut M, Kerle N, Poesen J, Hervás J (2012). Object-oriented identification of forested landslides with derivatives of single pulse LiDAR data. Geomo.

[25] Dabiri Z (2020). Assessment of landslide-induced geomorphological changes in Hítardalur Valley, Iceland, using Sentinel-1 and Sentinel-2 data. Appl. Sci..

[26] Bacha AS, Van Der Werff H, Shafique M, Khan H (2020). Transferability of object-based image analysis approaches for landslide detection in the Himalaya Mountains of northern Pakistan. Int. J. Remote Sens..

[27] Chen T, Trinder JC, Niu R (2017). Object-oriented landslide mapping using ZY-3 satellite imagery, random forest and mathematical morphology, for the Three-Gorges Reservoir, China. Remote Sens. Basel.

[28] Jiang X, Wang Y, Liu W, Li S, Liu J (2019). Capsnet, cnn, fcn: Comparative performance evaluation for image classification. Int. J. Mach. Learn. Comput..

[29] Martinez JAC, La Rosa LEC, Feitosa RQ, Sanches IDA, Happ PN (2021). Fully convolutional recurrent networks for multidate crop recognition from multitemporal image sequences. ISPRS J. Photogramm. Remote Sens..

[30] Mboga N (2019). Fully convolutional networks and geographic object-based image analysis for the classification of VHR imagery. Remote Sens. Basel.

[31] Chen Z, Zhang Y, Ouyang C, Zhang F, Ma J (2018). Automated landslides detection for mountain cities using multi-temporal remote sensing imagery. Sensors.

[32] Sameen MI, Pradhan B (2019). Landslide detection using residual networks and the fusion of spectral and topographic information. IEEE Access.

[33] Soares, L. P., Dias, H. C. & Grohmann, C. H. Landslide segmentation with U-Net: evaluating different sampling methods and patch sizes. arXiv preprint https://arxiv.org/abs/2007.06672 (2020).

[34] Liu P, Wei Y, Wang Q, Chen Y, Xie J (2020). Research on post-earthquake landslide extraction algorithm based on improved U-Net model. Remote Sens. Basel.

[35] Qi W, Wei M, Yang W, Xu C, Ma C (2020). Automatic mapping of landslides by the ResU-Net. Remote Sens. Basel.

[36] Su Z (2020). Deep convolutional neural network–based pixel-wise landslide inventory mapping. Landslides.

[37] Masoud KM, Persello C, Tolpekin VA (2020). Delineation of agricultural field boundaries from sentinel-2 images using a novel super-resolution contour detector based on fully convolutional networks. Remote Sens. Basel.

[38] Osanai N (2019). Characteristics of landslides caused by the 2018 Hokkaido Eastern Iburi Earthquake. Landslides.

[39] Kameda J (2019). Fluidized landslides triggered by the liquefaction of subsurface volcanic deposits during the 2018 Iburi-Tobu earthquake, Hokkaido. Sci. Rep..

[40] Yamagishi H, Yamazaki F (2018). Landslides by the 2018 Hokkaido Iburi-Tobu Earthquake on September 6. Landslides.

[41] Aimaiti Y, Liu W, Yamazaki F, Maruyama Y (2019). Earthquake-induced landslide mapping for the 2018 Hokkaido Eastern Iburi earthquake using PALSAR-2 data. Remote Sensing.

[42] Japan, G. S. I. G. O. *2018-Hokkaido Eastern Iburi Earthquake*. https://www.gsi.go.jp/BOUSAI/H30-hokkaidoiburi-east-earthquake-index.html#1 (2018).

[43] Zhang S, Li R, Wang F, Iio A (2019). Characteristics of landslides triggered by the 2018 Hokkaido Eastern Iburi earthquake, Northern Japan. Landslides.

[44] Lin C-W (2011). Landslides triggered by the 7 August 2009 Typhoon Morakot in southern Taiwan. Eng. Geol..

[45] Lin C-W (2013). Recognition of large scale deep-seated landslides in forest areas of Taiwan using high resolution topography. J. Asian Earth Sci..

[46] Hu X, Hu K, Tang J, You Y, Wu C (2019). Assessment of debris-flow potential dangers in the Jiuzhaigou Valley following the August 8, 2017, Jiuzhaigou earthquake, western China. Eng. Geol..

[47] Zhao B (2018). Landslides and dam damage resulting from the Jiuzhaigou earthquake (8 August 2017), Sichuan, China. R. Soc. Open Sci..

[48] Drusch M (2012). Sentinel-2: ESA's optical high-resolution mission for GMES operational services. Remote Sens. Environ..

[49] Sentinel, E. User Handbook. *ESA Standard Document*, Vol. 64.

[50] Long, J., Shelhamer, E. & Darrell, T. in *Proceedings of the IEEE Conference on Computer Vision and Pattern Recognition.* 3431–3440.

[51] Ronneberger, O., Fischer, P. & Brox, T. in *International Conference on Medical Image Computing and Computer-Assisted Intervention.* 234–241 (Springer).

[52] Zhang C (2020). Identifying and mapping individual plants in a highly diverse high-elevation ecosystem using UAV imagery and deep learning. ISPRS J. Photogramm. Remote. Sens..

[53] Zhu XX (2017). Deep learning in remote sensing: a comprehensive review and list of resources. IEEE Geosci. Remote Sens. Mag..

[54] Cui B, Fei D, Shao G, Lu Y, Chu J (2019). Extracting Raft aquaculture areas from remote sensing images via an improved U-Net with a PSE structure. Remote Sens. Basel.

[55] Mohammadimanesh F, Salehi B, Mahdianpari M, Gill E, Molinier M (2019). A new fully convolutional neural network for semantic segmentation of polarimetric SAR imagery in complex land cover ecosystem. ISPRS J. Photogramm. Remote. Sens..

[56] Abderrahim, N. Y. Q., Abderrahim, S. & Rida, A. in *2020 IEEE International Conference of Moroccan Geomatics (Morgeo).* 1–4 (IEEE).

[57] Khryashchev, V. & Larionov, R. in *2020 Moscow Workshop on Electronic and Networking Technologies (MWENT).* 1–5 (IEEE).

[58] Zhang Z, Liu Q, Wang Y (2018). Road extraction by deep residual u-net. IEEE Geosci. Remote Sens. Lett..

[59] He, K., Zhang, X., Ren, S. & Sun, J. in *Proceedings of the IEEE Conference on Computer Vision and Pattern Recognition.* 770–778.

[60] Kingma, D. P. & Ba, J. Adam: A method for stochastic optimization. arXiv preprint https://arxiv.org/abs/1412.6980 (2014).

[61] Yi Y, Zhang W (2020). A new deep-learning-based approach for earthquake-triggered landslide detection from single-temporal RapidEye satellite imagery. IEEE J. Sel. Top. Appl. Earth Obs. Remote Sens..

